# Graft nephrectomy in children

**DOI:** 10.1007/s00467-017-3697-1

**Published:** 2017-06-19

**Authors:** Benedict L. Phillips, Chris J. Callaghan

**Affiliations:** Department of Nephrology and Transplantation, Guy’s Hospital and the Evelina London Children’s Hospital, London, UK

**Keywords:** Pediatric, Renal, Transplant, Graft nephrectomy

## Abstract

Kidney transplantation is recognised as the gold standard treatment of end-stage renal disease in most children, with excellent graft survival rates. When graft failure occurs, renal transplant recipients (RTRs) have the option of removal of the transplant (graft nephrectomy [GN]), or leaving the failed transplant in situ. The aims of this review are to discuss the indications for GN, surgical techniques, outcomes after GN (including risks of allosensitisation and the impact on subsequent transplants), and the possible role of routine GN in the asymptomatic RTR with a failed renal allograft. Literature in both the pediatric and adult renal transplant fields is reviewed. We also discuss how future research in this area could advance our knowledge of which patients to select for GN, and the most appropriate surgical approach.

## Introduction

Renal transplantation is widely recognised as the gold standard treatment of end-stage renal disease in children. Graft survival rates are excellent, with pediatric renal transplant recipients (RTRs) from living donors and deceased donors having 5-year graft survival of 83% and 71% respectively [1]. However, 20% of children with a renal transplant have a failing graft, most commonly due to chronic rejection (36%), acute rejection (13%), or vascular thrombosis (7%) [1]. Unlike recipients of failing heart, lung, or liver transplants, RTRs with failing grafts have access to a long-term form of organ support, i.e. dialysis. As a result, RTRs have the option of leaving a failed allograft in situ.

The alternative is surgical removal of the failed graft (graft nephrectomy [GN]). Previous studies have shown that more than half of children with failed renal allografts undergo GN [2, 3], suggesting that GN occurs frequently in the pediatric RTR population. Children with graft failure within a year of transplantation are four times more likely to undergo GN [2]. However, relatively little has been written in the pediatric literature on this subject, and to our knowledge, only one national guideline has been published [4]. In addition, it is not known whether or not failed renal allografts should be routinely removed, i.e. in asymptomatic RTRs. This is of particular importance in pediatric RTRs because of the need to avoid HLA sensitisation, and therefore optimise the child’s opportunity to receive further renal transplants in the future [5].

The aim of this review is therefore to examine the role of GN in children. This review focuses on the indications for GN, surgical techniques (including non-surgical approaches to graft devascularisation), outcomes after GN, risks of allosensitisation after GN, and the impact on subsequent outcomes after re-transplantation. Also, we discuss the management of immunosuppression after GN. Finally, we consider the possible role of routine GN in the RTR with a failed graft, and the direction of future research in this area.

## Indications

Widely accepted indications for GN in children can be divided into absolute and relative groups. Absolute indications include:

1. Unsalvageable acute venous graft thrombosis: GN should be performed to prevent graft rupture and catastrophic bleeding.
2. Unsalvageable acute arterial graft thrombosis: GN should be performed to prevent the high risk of graft necrosis and subsequent infection that is likely to occur in the absence of a collateral blood supply to the kidney transplant.
3. Graft malignancy not appropriate for treatment with less invasive strategies, such as partial nephrectomy [6, 7], radiofrequency ablation [7] or cryotherapy [8].

Relative indications for GN include:

1. Localising signs and/or symptoms (e.g. haematuria, graft pain) indicating a chronic alloimmune response in a failed or failing renal transplant. Some patients may also have non-specific malaise, increased inflammatory markers, and erythropoietin resistance [9]. Other causes of these features should be excluded before considering GN, e.g. with imaging, urine culture, cystoscopy etc.
2. Recurrent or severe graft pyelonephritis, not responding to appropriate antimicrobial therapy.
3. To enable complete withdrawal of immunosuppression, e.g. in post-transplant lymphoproliferative disorder non-responsive to standard treatment [10], or persistent BK nephropathy resistant to reduced immunosuppression and anti-virals [11].
4. To create space for re-transplantation: cross-sectional imaging is usually required to define the anatomy and provide a more objective analysis of the space available for a re-transplant. GN may be performed at the time of transplantation, or before listing.

## Surgical technique

There are three recognised surgical techniques for GN: intraperitoneal; extraperitoneal with an intra-capsular (IC) approach; or extraperitoneal with an extra-capsular (EC) approach.

The favoured technique is determined by multiple factors, including the original method of graft implantation, the timing of GN after transplantation, the possible presence of intraperitoneal disease, and the surgeon’s preference.

**Fig. 1:**
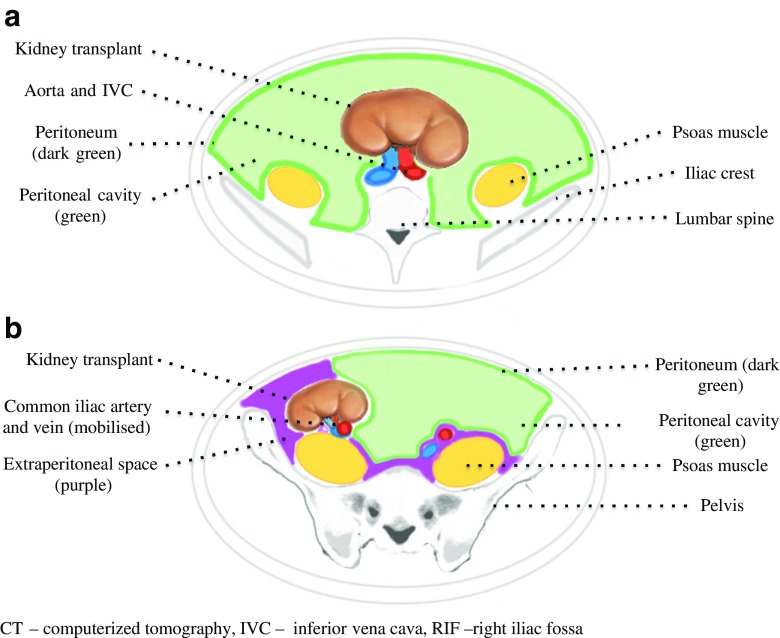
**a** Cross-section of the abdomen as seen on CT. Intraperitoneal kidney transplant. The kidney is implanted into the peritoneal cavity (*shaded in green*) and is anastomosed on the aorta and inferior vena cava (*IVC*). **b** Cross-section of the pelvis as seen on CT. Extraperitoneal kidney transplant. The kidney is implanted in the right iliac fossa (RIF), outside of the peritoneum (marked in *dark green*), within the extraperitoneal space (*shaded in purple*)

Pediatric RTRs weighing less than 15–20 kg at the time of transplantation often undergo intraperitoneal kidney implantation, although approaches vary among units (Fig. 1a) [12, 13]. This enables adequate access to larger recipient vessels (e.g. the aorta and inferior vena cava) and the necessary space for the graft to lie in. Intraperitoneal kidney grafts can only be explanted via an intraperitoneal approach, i.e. entering into the peritoneal cavity.

**Fig. 2:**
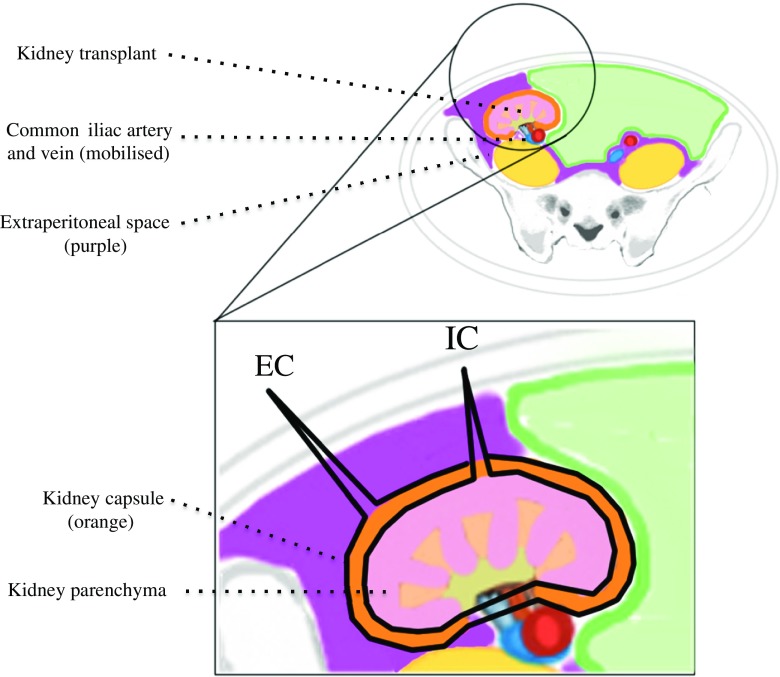
Cross-section of the abdomen of a RIF kidney transplant in the extraperitoneal space. The extra-capsular (*EP*) and intra-capsular (*IC*) approaches to graft nephrectomy are marked in* black*

Pediatric RTRs with grafts implanted into the extraperitoneal space account for the majority of cases, typically in children weighing more than 15–20 kg at the time of transplantation (Fig. 1b). Extraperitoneal implantation is generally preferred in larger children to avoid the complications associated with entering the peritoneal cavity (e.g. visceral injury, adhesions), to access extraperitoneal structures such as the iliac blood vessels and bladder, and for the ease of subsequent graft biopsy. GN in this patient group may be performed via either intraperitoneal or extraperitoneal (IC or EC) approaches (Fig. 2). It is important to consider the risks and benefits of each technique.

Following extraperitoneal transplantation, the capsule of the kidney becomes progressively adherent to extra-peritoneal tissues. If GN is performed within 2–6 weeks of transplantation, the kidney can usually be explanted in its entirety (EC approach). After 2–6 weeks post-transplantation, there is no clear plane between native tissues and the allogeneic renal capsule, making an EC approach increasingly challenging. Therefore, removal of the extraperitoneal graft is usually achieved by bluntly separating the renal capsule from the underlying parenchyma (IC approach; Fig. 2) [14]. With the IC approach, it is more difficult to access the graft vessels and ureter, leading to ligation nearer the graft hilum, and resulting in more allogeneic tissue being left in situ. Alternatively, the surgeon may enter the peritoneal cavity to then excise a graft placed extraperitoneally; this is also an intraperitoneal approach. Better access to the donor blood vessels and ureter is therefore possible. Removing the ureter and blood vessels entirely is preferable to avoid allosensitisation, but exposes the patient to increased surgical risk, including longer operating times, bladder leaks, need for post-operative bladder catheterisation, vascular reconstruction and its complications.

Each of the three surgical approaches described above has its advantages and disadvantages (Table 1). These techniques are almost always performed as open surgery, although laparoscopic GN of a graft placed intraperitoneally has been described [15].

**Table 1:** The different surgical techniques for graft nephrectomy (GN), with relative advantages and disadvantages

Approach	Advantages	Disadvantages
Intraperitoneal	Only feasible access to intraperitoneal grafts More complete removal of allogeneic tissues Likely reduced rate of lymphoceles and collections	Risk of damaging intraperitoneal structures (e.g. bowel) Risk of post-operative ileus Risk of complications from bowel adhesions (e.g. obstruction)
Extra-capsular	More complete removal of allogeneic tissues Easy plane in the early post-transplant phase Good access to the blood vessels and bladder Avoidance of the bowel	Challenging plane after the early post-transplant phase Risk of lymphoceles and collections
Intra-capsular	Accessible plane after the early post-transplant phase Avoidance of bowel	Less complete removal of allogeneic tissues Possible increased blood loss from the bare parenchyma More difficult access to the blood vessels and bladder

## Non-surgical devascularisation

Renal artery embolisation (RAE) is a minimally invasive alternative to GN and has been used in both adults and children [16–19]. Devascularisation of the allograft is achieved by percutaneous puncture of the femoral artery, followed by induction of graft thrombosis using ethanol, stainless steel coils, polyvinyl alcohol or tris acryl microspheres. The aim of RAE is complete infarction of the allograft; however, incomplete thrombosis of the graft occurs in between 15 and 35% of cases [20–23].

RAE may be advantageous over GN as it appears to reduce the risk of some of the complications associated with open surgery, including blood loss and requirement for blood transfusion [24]. RAE is also associated with reduced pain and length of stay in hospital [18]. However, RAE can be associated with other complications, such as post-embolisation syndrome (fever, graft pain, haematuria) and necrotic pyelonephritis, both affecting approximately 10% of patients [17]. These graft-related complications can be avoided by using RAE as a “neo-adjuvant” intervention, one or two days before GN. This combined approach appears to reduce the intraoperative blood loss [24–26] and operative time of GN [24, 26]. Less common complications of RAE include arterial pseudoaneurysm [27] and coil migration with limb embolisation [28].

At present, RAE is not widely used, and the patient groups most likely to benefit from RAE, GN, or combined RAE-GN, are as yet poorly defined in the pediatric RTR population. RAE alone may be most useful in patients with a prohibitively high operative risk (e.g. severe cardiorespiratory disease), with combined RAE-GN reserved for those at a significantly increased risk of intraoperative bleeding [4].

## Outcomes after GN

Outcomes after GN can be analysed on the basis of patient age group (pediatric or adult), surgical approach (intraperitoneal, or extraperitoneal EC/IC), or timing of surgery (early or late post-transplant). Outcomes may be related directly to the surgery in the short-term, or may be immunological with long-term sequelae.

### Short-term morbidity and mortality

The literature on outcomes after GN contains many retrospective analyses, but most are limited by their study design. In addition, many reports do not specify which GN surgical technique was used. No randomised controlled trials (RCTs) are available.

In children, only two papers have been published on outcomes after GN. The largest analysis (*n* = 53) was performed by Zerouali et al. [3]. Children undergoing GN within a month post-transplant had surgery via the EC approach (*n* = 19); all others had grafts removed via the IC approach (*n* = 44). Complications were not stratified according to the surgical approach. There were no early post-operative deaths following GN, although the follow-up period was not defined. Twenty per cent of children developed complications after GN, including wound infection (9%), deep vein thrombosis (5%) and chest infection (2%). Median length of stay in hospital was 19.5 days; no comment was made on the need for blood transfusions. It should be noted that this study was performed on GNs between 1977 and 1999, i.e. before the modern immunosuppression era. In a more recent analysis of 18 children who underwent GN, none of the patients had major intra- or post-operative complications requiring a secondary operation, unplanned intensive care admission or blood transfusion within the first 14 days [2]. The surgical approach was not described.

In adults, the largest study examining outcomes after GN in adults was published by Johnston et al. [29], who used the US Renal Data System (RDS) database. Of the 6,213 adults who underwent GN between 1995 and 2003, the reported mortality rate was 1% during hospital admission, and 5% within 90 days of surgery. Death was more common in patients undergoing nephrectomy for graft failure within a year of transplantation, presumably because of the heavier immunosuppressive burden during this period. Sepsis was the most common complication of GN, affecting 6% during their hospital stay and 11% within 90 days of GN. There have been a number of other retrospective analyses examining early post-operative outcomes following GN in adults, with a complication rate of 20–60%, and a mortality rate of 0–7% [30–33]. Sepsis appears to be the most common complication.

There are no studies comparing IC and EC GN techniques in children, and only small analyses in adults. Vavallo et al. [34] examined the outcomes of 89 adults undergoing GN; 51 by EC GN and 32 by IC GN. Overall, 9 patients (10%) had bleeding, with 6 (7%) having infection/sepsis, and 4 (4%) developing a lymphocele. Two patients needed surgery for arterial damage. No deaths were reported. Surprisingly, mean operating time, blood loss, need for blood transfusion and perioperative complication rates of the two techniques were similar. Overall, 10% of patients required a blood transfusion. The only difference observed between the outcomes after the two techniques was mean hospital length of stay, which was 13.8 days in the EC group and 7.6 days in the IC group (*p* = 0.01). Smaller studies comparing the IC and EC techniques have not shown a clear association between technique and blood loss [35–37].

Major vascular complications occur in approximately 5% of cases and are associated with poor outcomes [38]. Complications include major haemorrhage requiring ligation of the external iliac artery, and pseudoaneurysm formation [38–41].

Although GN is associated with short-term morbidity and mortality, there is some evidence that long-term mortality in patients who undergo GN is less than those with a failed renal allograft who have not had a GN. A large analysis of the US RDS registry between 1994 and 2004 showed that, of 10,915 RTRs with failed grafts on dialysis, 31.5% underwent GN, which was associated with a reduced relative risk of death from all causes when compared with those who had not had GN. This difference persisted after risk adjustment for socioeconomic factors and co-morbidity burden (adjusted hazard ratio 0.68; 95% confidence interval 0.63–0.74) [42].

In summary, GN is major surgery, but post-operative mortality appears to be very low in children, with reasonable rates of major complications. As expected, mortality rates after GN in adults are higher. There is no strong evidence favouring one surgical approach over another with regard to short-term morbidity and mortality. Whichever surgical approach is used, careful surgery with meticulous haemostasis is advised [4].

### Allosensitisation and subsequent graft outcomes

The avoidance of sensitisation to HLA (allosensitisation) is a critical issue in renal transplantation, particularly pediatric renal transplantation, where patients are expected to need more than one graft in their lifetime. Allosensitisation occurs through exposure to non-self HLA via a blood transfusion, transplantation, or pregnancy, resulting in the production of antibodies directed against HLA. The immunological pathways of HLA-Ab production have been fully described elsewhere [5]. The presence of HLA antibodies is associated with an increased risk of rejection and graft loss in current and subsequent transplants. After graft failure, broad HLA sensitisation (high panel reactive antibody [PRA]) results in reduced access to the donor pool, with longer waiting times and the potential morbidity and mortality associated with prolonged dialysis.

The impact of GN on allosensitisation and subsequent graft outcomes is difficult to determine because of multiple potential confounding factors. Most importantly, one of the major indications for GN is symptomatic graft rejection, implying that the recipient is sensitised before GN. In addition, GN may lead to the need for blood transfusion, and therefore the surgical technique and variation in transfusion practice will also have an impact on sensitisation. Finally, the management of immunosuppressant withdrawal after GN is not standardised, is poorly described in most papers, and is also highly likely to influence subsequent sensitisation after GN [43]. Examining change in PRA over the GN period attempts to control for some of the above factors, but may not take into account the potential absorptive capacity of the graft (“sponge hypothesis” [44]), which may also act as a confounding variable in retrospective analyses. Leaving allogeneic tissue in situ is more likely with the IC approach, and may also influence allosensitisation. These caveats should be kept in mind when considering the literature.

In pediatric RTRs, Minson et al. [2] performed a retrospective analysis of 34 children who had renal allograft failure. PRA was significantly higher for children following GN (mean PRA 87.9%) compared with children who had not undergone GN (65.2%; *p* = 0.0003). However, the relative change in PRA following GN was not recorded, meaning that the possibility that the GN group may have had higher PRA than the non-GN group even before the operation cannot be excluded. Indeed, the GN group had a higher incidence of biopsy-proven Banff II acute rejection before undergoing GN (*p* = 0.04). Zerouali et al. did not observe any difference in PRA score between children who had GN (*n* = 63) and those who did not (*n* = 82) [3].

There have been two studies in adults indicating that the timing of GN is an important factor in allosensitisation. Sener et al. [45] found that patients who had GN following early graft failure (<6 months) demonstrated a reduction in PRA score by 19% (*p* = 0.02), whereas PRA score increased following late GN. Studies that did not take into account the timing of GN showed that GN was associated with an increase in detectable HLA-Ab and new DSA [46–50]. Analyses comparing IC and EC GN on allosensitisation were unable to detect a difference in PRA score between the two techniques [35, 37].

The impact of GN on subsequent renal transplant outcomes is similarly ill-defined. There are no studies in pediatric RTRs, but in adults, Johnston et al. [29] found that patients undergoing GN following early graft failure (<12 months) were at a lower risk of graft failure once retransplanted (HR 0.72, 95% CI 0.56–0.94). In contrast, patients who had GN following late graft failure were at a significantly higher risk of subsequent graft failure (HR 1.20, 95% CI 1.02–1.41).

One meta-analysis of PRA and subsequent graft outcomes after GN has been published by Wang et al. [51]. Eight retrospective analyses were identified, but only papers published in open access journals were included. The meta-analysis showed that patients undergoing GN had no difference in one-year graft or patient survival compared with those being re-transplanted without GN. GN was associated with a significantly higher increase in PRA score compared with the non-GN group (odds ratio 1.62, 95% confidence interval 1.17–2.23). It should be noted that Wang’s criterion for entry into the meta-analysis was asymptomatic non-functioning renal allografts (“routine GN”); however, of the 8 papers, 6 included GN performed for symptoms. The inclusion of non-randomised observational studies introduces the possibility that the confounding factors discussed above might influence outcomes, and therefore it is difficult to draw meaningful conclusions from these results.

### Immunosuppression after GN

The management of immunosuppression after GN in children is a complex area, with little evidence supporting practice. Even in the adult field, the evidence base is limited.

Withdrawal of immunosuppression is an independent predictor of allosensitisation after GN [46, 52]; this is not surprising given that allogeneic tissue is likely to be present after GN, as discussed above. There is therefore some rationale for maintaining immunosuppression following GN; however, this needs to be balanced with the risks of remaining on immunosuppression whilst on dialysis (e.g. infection [53, 54], cardiovascular disease and malignancy). Current guidelines recommend that all immunosuppression, apart from steroids, should be stopped immediately after GN [4]. There should be subsequent gradual withdrawal of steroids.

However, these guidelines were aimed primarily at adult RTRs and considerations with pediatric RTRs are obviously different, as children are more likely to have live donors available after graft loss, and they do not wait as long on the deceased donor waiting list as adults. We would therefore suggest that the risk of HLA sensitisation might be minimised after GN in children by maintaining immunosuppression with at least two agents. This is based on studies in adults that identified prolonged immunosuppression as a protective factor in reducing antibody levels after graft failure [50, 55]. This approach to immunosuppression management after GN should be modified for children at a high risk of infection or cancer whilst on dialysis, or in those countries where deceased donor waiting times for children are prolonged (e.g. more than 1 year).

## Is there a role for routine GN?

Routine GN can be defined as GN performed for a failed graft in an asymptomatic RTR, i.e. without the pathological conditions discussed in the section 'Indications' above. Routine GN does not appear to be widely practised, with less than 5% of transplant surgeons in the USA carrying these out in adult RTRs [56].

Del Bello et al. described their experience of performing routine (“systematic”) GN in 17 adult RTRs whose grafts had failed [46]. Patients in this study had all immunosuppressants (other than steroids) stopped when their grafts failed and dialysis was started; steroids were ceased 6 months after dialysis was initiated. The timing of routine GN after dialysis and surgical technique were not described. After routine GN, average hospital stay was 6 days, and 30% of patients had a complication during their stay; 35% required a blood transfusion. Del Bello et al. showed that there was no difference in the emergence of donor-specific HLA antibodies (class I or II) in the routine GN group compared with a group of patients having GN for symptoms/signs (*n* = 31). Patients from a historical cohort who had not undergone GN, but had the same immunosuppressive withdrawal regimen, had lower DSAs at the last follow-up than the routine GN group (52% vs 82%). This study was non-randomised and many centres would have continued immunosuppressants for longer after graft failure. The impact of routine GN on fluid balance and inter-dialytic weight gains were not assessed.

Therefore, there is no strong evidence to support the use of routine GN at present. The decision to perform GN should be made having considered the risks and benefits, and on a case-by-case basis.

## Directions of future research

The current literature on GN in pediatric RTRs is, in general, difficult to interpret, as the number of reports is small. Even in adults, the evidence base is limited, and many papers have insufficient information on the timing of GN, the surgical technique used, and the immunosuppression withdrawal strategy after graft failure or GN. Future analyses should contain detailed information on the above to provide an evidence base to guide clinicians caring for children with failed renal allografts.

The only feasible way of definitively determining the role of routine GN in clinical practice is by carrying out an RCT. This should be performed in adults first, owing to greater patient numbers, ethical considerations, and given the complexities of intra-peritoneal graft removal and the relatively higher chance of blood transfusion with pediatric surgery. If completed in adults, trials will be required in children given the high requirement for future re-transplantation in the pediatric renal failure population.

## Summary and conclusions

There are widely-accepted absolute and relative indications for GN. Routine GN for failing grafts without these indications is not commonly practiced, and there is little evidence to support this approach. The three main surgical techniques for GN have been defined. Although there is no clear evidence to support one approach over the other, we suggest that as much allogeneic tissue should be removed as possible, to minimise the risk of subsequent HLA sensitisation. This is of particular importance in children. Meticulous surgical technique to avoid blood transfusion is also essential. RAE may be used as an alternative to, or in a “neo-adjuvant” intervention with, GN. RAE alone may be most useful in patients with a high operative risk or used before GN to reduce intraoperative blood loss and operative time.

The mortality rate of GN in children appears to be very low; complications affect 20% of children, with sepsis being the most common complication. The impact of GN on allosensitisation and subsequent graft outcomes is difficult to determine because of multiple potential confounding factors. Indeed, children undergoing GN are likely to have a raised PRA score before the operation as the indication for GN is often rejection. Furthermore, weaning of immunosuppression leads to sensitisation independently of GN [52]. It is therefore not possible to definitively determine the causality of raised PRA score in this setting. The impact of GN on subsequent renal transplant outcomes is similarly ill-defined in children. However, in adults, GN following early graft failure appears to be associated with better subsequent graft survival in the re-transplanted kidney, and worse graft survival following late GN.

Studies reporting outcomes after GN should report sufficient variables to enable meaningful comparisons with others in the field. RCTs are needed to address the role of routine GN in the adult and pediatric RTR populations.

## Key summary points

- The mortality rate of GN in children appears to be very low, with complications affecting approximately 20% of children.
- There are three main surgical techniques for performing GN and there is no clear evidence to support one approach over the others.
- As much allogeneic tissue should be removed as possible during GN to minimise the risk of subsequent HLA sensitisation.
- RAE may be used as an alternative to, or “neo-adjuvant” intervention with, GN.
- Routine GN for the failing graft in an asymptomatic RTR is not commonly practiced.

## Multiple choice questions (answers are provided following the reference list)

1. Regarding the surgical techniques of graft nephrectomy, which of the following statements is FALSE?
  - All intraperitoneal grafts are explanted via the intraperitoneal approach.
  - The extra-capsular approach is preferred soon after transplantation, as the surrounding tissue has not yet become adherent to the capsule of the graft.
  - The intra-capsular approach is preferred late after transplantation as little allogeneic tissue is left in situ.
  - Renal artery embolisation may be used before graft nephrectomy to reduce intraoperative bleeding and transfusion requirements.
2. Regarding the indications for graft nephrectomy, which of the following statements is FALSE?
  - Unsalvageable acute arterial and/or venous graft thromboses are absolute indications for graft nephrectomy.
  - Minimally invasive strategies, such as renal artery embolisation, should be considered first for graft malignancy, before graft nephrectomy.
  - Graft nephrectomy can be performed at the time of retransplantation.
  - BK nephropathy is a relative indication for graft nephrectomy, if antiviral treatments have been unsuccessful.
3. Regarding allosensitisation, which of the following are NOT considered to be likely sensitising events?
  - Blood transfusion
  - Pregnancy
  - Transplantation
  - Plasma exchange

## Abbreviations

DSA: Donor-specific antibody
EC: Extra-capsular
GN: Graft nephrectomy
IC: Intra-capsular
RAE: Renal artery embolisation
RTR: Renal transplant recipient
RDS: Renal data system
